# Innate Immune Suppression Enables Frequent Transfection with RNA Encoding Reprogramming Proteins

**DOI:** 10.1371/journal.pone.0011756

**Published:** 2010-07-23

**Authors:** Matthew Angel, Mehmet Fatih Yanik

**Affiliations:** 1 Department of Electrical Engineering and Computer Science, Massachusetts Institute of Technology, Cambridge, Massachusetts, United States of America; 2 Department of Biological Engineering, Massachusetts Institute of Technology, Cambridge, Massachusetts, United States of America; University of Georgia, United States of America

## Abstract

**Background:**

Generating autologous pluripotent stem cells for therapeutic applications will require the development of efficient DNA-free reprogramming techniques. Transfecting cells with in vitro-transcribed, protein-encoding RNA is a straightforward method of directly expressing high levels of reprogramming proteins without genetic modification. However, long-RNA transfection triggers a potent innate immune response characterized by growth inhibition and the production of inflammatory cytokines. As a result, repeated transfection with protein-encoding RNA causes cell death.

**Methodology/Principal Findings:**

RNA viruses have evolved methods of disrupting innate immune signaling by destroying or inhibiting specific proteins to enable persistent infection. Starting from a list of known viral targets, we performed a combinatorial screen to identify siRNA cocktails that could desensitize cells to exogenous RNA. We show that combined knockdown of interferon-β (*Ifnb1*), *Eif2ak2*, and *Stat2* rescues cells from the innate immune response triggered by frequent long-RNA transfection. Using this technique, we were able to transfect primary human fibroblasts every 24 hours with RNA encoding the reprogramming proteins Oct4, Sox2, Klf4, and Utf1. We provide evidence that the encoded protein is active, and we show that expression can be maintained for many days, through multiple rounds of cell division.

**Conclusions/Significance:**

Our results demonstrate that suppressing innate immunity enables frequent transfection with protein-encoding RNA. This technique represents a versatile tool for investigating expression dynamics and protein interactions by enabling precise control over levels and timing of protein expression. Our finding also opens the door for the development of reprogramming and directed-differentiation methods based on long-RNA transfection.

## Introduction

Somatic cells can be reprogrammed to a pluripotent-stem-cell state by maintaining expression of specific combinations of proteins through several rounds of cell division [1]–[7]. Although methods of reprogramming human somatic cells using non-viral DNA vectors have been reported [8], [9], the risk of genomic disruption may limit the therapeutic potential of these techniques. Reprogramming by direct protein transduction has also been demonstrated [10], [11], however reprogramming human cells using recombinant proteins is currently an inefficient process. We postulated that expressing reprogramming proteins by repeated transfection with protein-encoding RNA could avoid the limitations of both DNA- and protein-based reprogramming techniques, however we discovered that long RNA triggers a potent innate immune response in human cells, even when the RNA is capped and polyadenylated to mimic eukaryotic mRNA. To solve this problem, we developed a method of suppressing innate immunity to enable frequent transfection with protein-encoding RNA.

The mechanisms by which cells distinguish endogenous RNA from the exogenous RNA produced during viral infection are the subject of ongoing investigation and debate [12]–[18]. In humans, exogenous RNA is a pathogen-associated molecular pattern (PAMP) for which toll-like receptor 3,7/8 (Tlr3,7*/*8) [19]–[22], and members of the Rig1 receptor family [12] are pattern-recognition receptors (PRRs). Once activated, these PRRs initiate cascades of intracellular signaling that result in upregulation of PRRs, hypersensitizing cells to subsequent exposure to exogenous RNA. PRR activation also results in the production of type I interferons, which hypersensitize nearby cells. In addition, long RNA binds and activates Eif2ak2, blocking translation of both exogenous and endogenous RNA [23], [24]. Although the innate immune response to exogenous RNA is initiated and regulated by intra- and extracellular signaling networks containing a great deal of redundancy, RNA viruses have evolved methods of disrupting these pathways by destroying or inhibiting specific immune-related proteins to enable persistent infection [25]. We hypothesized that mimicking viral immunoinhibition by co-transfecting cells with an siRNA cocktail designed to directly knock down expression of immune-related proteins could desensitize cells to exogenous RNA, and thus enable repeated long-RNA transfection.

## Results

**Figure 1:**
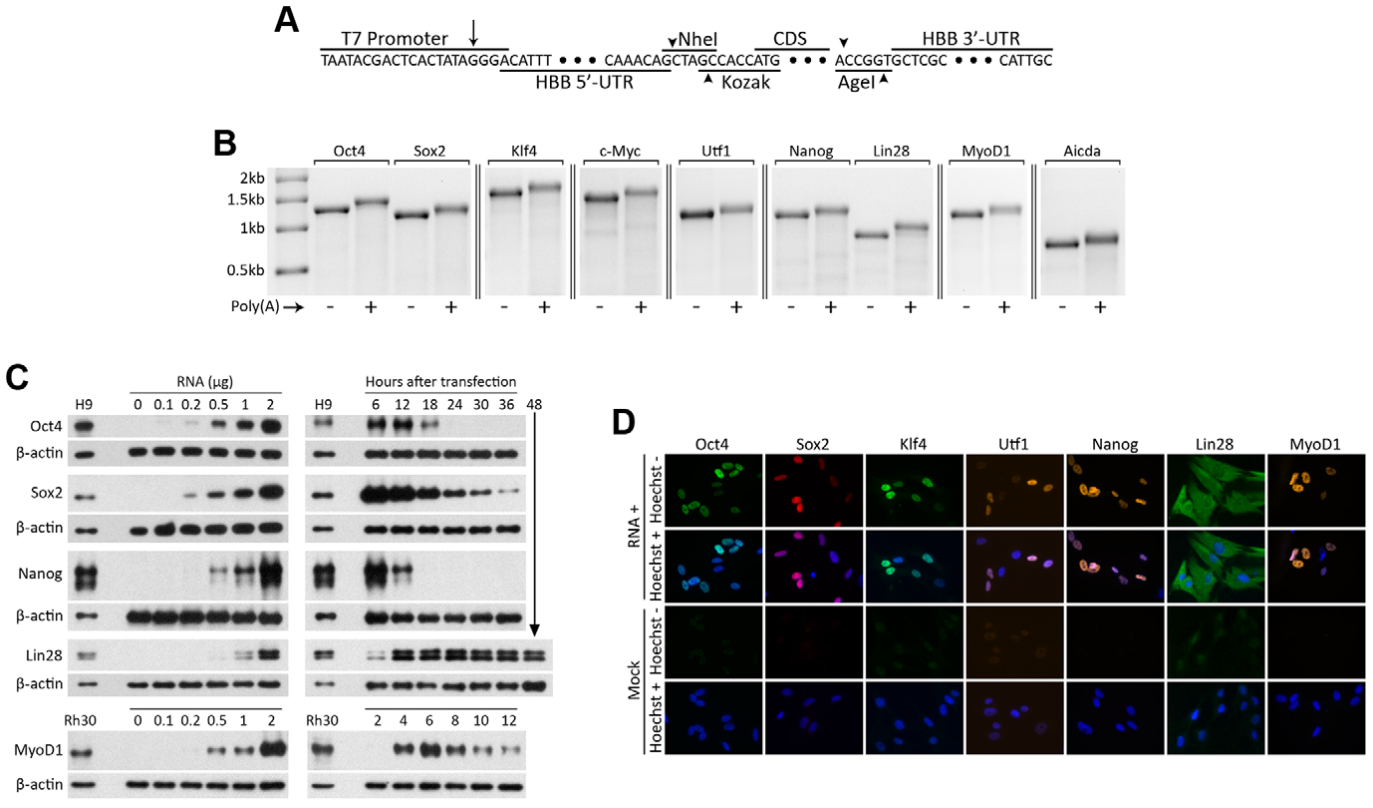
Long-RNA transfection yields ES-cell-level expression of reprogramming proteins in primary human fibroblasts. A. The transcribed strand of an *Hbb*-UTR-stabilized in vitro-transcription template encoding an arbitrary protein. The long arrow indicates the first transcribed base, and short arrows indicate restriction-enzyme cleavage sites. B. In vitro-transcribed RNA encoding reprogramming proteins. C. Western blots showing expression levels and lifetimes of Oct4, Sox2, Nanog, Lin28, and MyoD1 proteins in MRC-5 human fetal lung fibroblasts transfected with protein-encoding RNA, relative to levels in hES (H9) and rhabdomyosarcoma (Rh30) cells. β-actin was used as a loading control. Left panels: The amount of RNA per 50 µL electroporation volume was varied as indicated. Cells were lysed 6 hours after transfection. Right panels: Cells were transfected with 1 µg of RNA, and lysed at the indicated times. D. Expression and nuclear localization of Oct4, Sox2, Klf4, Utf1, Nanog, Lin28, and MyoD1 protein following long-RNA transfection. Cells were fixed and stained 6–12 hours after transfection. For each protein, identical camera settings and exposure times were used for the RNA-transfected and mock-transfected samples.

**Figure 2:**
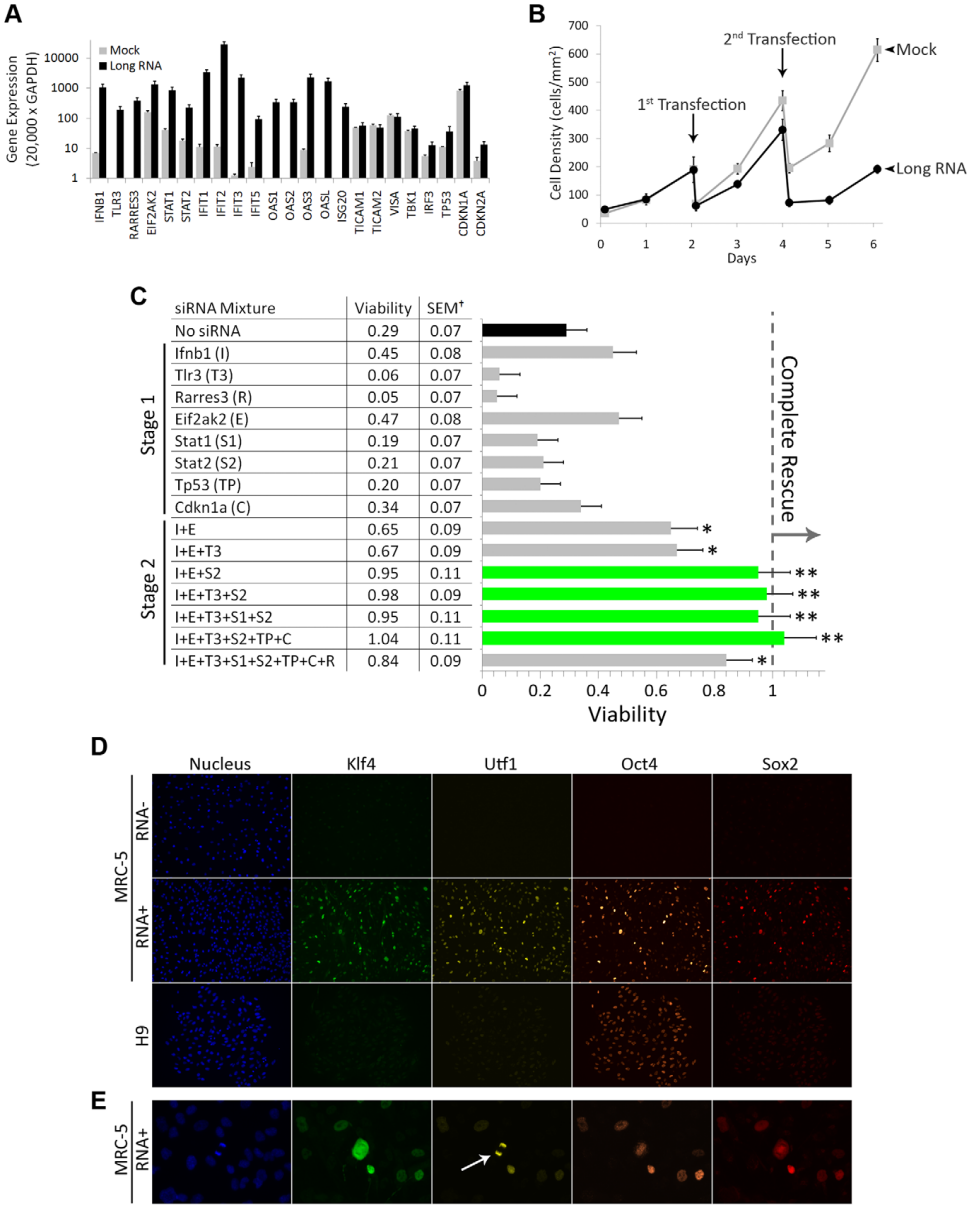
Innate immune suppression enables frequent long-RNA transfection. Combinatorial siRNA screening identifies siRNA cocktails that rescue cells from the innate immune response triggered by long-RNA transfection. A. Upregulation of innate immune genes following long-RNA transfection. MRC-5 fibroblasts were transfected with 0.4 µg of RNA per well of a 24-well plate using lipids. Expression of innate immune genes was measured by quantitative RT-PCR 24 hours after transfection. *Gapdh* was used as a loading control. Error bars indicate the standard deviation of replicate samples. B. Repeated long-RNA transfection causes cell death in human fibroblasts. MRC-5 fibroblasts were electroporated twice with 0.5 µg/50 µL of Lin28-encoding RNA at 48-hour intervals. Samples of cells transfected with RNA (black circles) and mock-transfected cells (gray squares) were trypsinized and counted at the indicated times. Data points and error bars indicate the mean and standard error of two independent experiments. Data points are connected for clarity. C. Combined knockdown of *Ifnb1*, *Eif2ak2*, and *Stat2* rescues cells from the innate immune response triggered by frequent long-RNA transfection. MRC-5 fibroblasts were transfected as in (B), but with the indicated siRNA on day 0, and 0.5 µg of Lin28-encoding RNA and additional siRNA on days 2 and 4 (Table S1). Samples of cells were trypsinized and counted 24 hours after the second long-RNA transfection (day 5). Values indicate cell count relative to mock-transfected cells. ^†^Standard error of replicate samples (n = 4). *p<0.05, **p<0.005. D. Frequent transfection of primary human fibroblasts with a mixture of RNA encoding the reprogramming proteins Oct4, Sox2, Klf4, and Utf1 yields sustained, ES-cell-level expression. Cells were reverse transfected with an immunosuppressive siRNA cocktail (Lipofectamine RNAiMAX, Invitrogen), and then transfected with protein-encoding RNA (0.1 µg of RNA per factor per well) using lipids every day for three days. Cells were fixed and stained 8 hours after the last transfection. For each protein, identical camera settings and exposure times were used for the mock-transfected, RNA-transfected, and hES-cell samples. E. Cells expressing reprogramming proteins undergo mitosis. Cells were transfected as in (D). Utf1 localized to chromosomes in mitotic cells (arrow).

We synthesized capped, polyadenylated transcripts containing the β-globin (*Hbb*) 5′- and 3′-untranslated regions (UTRs) [26]–[29] and the Oct4, Sox2, Klf4, c-Myc, Utf1, Nanog, Lin28, MyoD1, and Aicda coding sequences (Fig. 1A,B), and transfected human adult-dermal and fetal-lung fibroblasts using both electroporation and lipid-based transfection reagents. Electroporating cells with 1 µg of each RNA (in a 50 µL total volume) resulted in protein expression at or above ES-cell levels within 6 hours for Oct4, Sox2, and Nanog (Fig. 1C), and immunostaining showed correct sub-cellular localization (Fig. 1D). Transfected cells quickly upregulated many genes involved in the immune response to viral RNA including *Ifnb1*, *Tlr3*, *Rarres3*, *Eif2ak2*, *Stat1*,*2*, *Ifit1*,*2*,*3*,*5*, *Oas1*,*2*,*3*,*L*, and *Isg20* (Fig. 2A). Performing a second transfection after 48 hours resulted in significant cell death (Fig. 2B).

We previously showed that combined, siRNA-mediated knockdown of immune-related proteins could rescue primary human fibroblasts from the cell death caused by frequent transfection with protein-encoding in vitro-transcribed (ivT) RNA [30]. However, effective combinations all included siRNA targeting p53, suggesting incomplete immune suppression. To identify more effective immunosuppressive siRNA mixtures, we conducted a two-stage combinatorial siRNA screen starting from a list of known viral targets (Fig. 2C). In both stages, fibroblasts were electroporated with siRNA three times at 48-hour intervals (Table S1). RNA encoding Lin28 was included in the second and third transfections. Cells were counted 24 hours after the last transfection to assess viability. In the first stage, we transfected cells with siRNA targeting a single gene from the following list: *Ifnb1*, *Tlr3*, *Rarres3*, *Eif2ak2*, *Stat1*, *Stat2*, *Tp53*, and *Cdkn1a*. The highest viability was observed in cultures transfected with siRNA targeting either *Ifnb1* or *Eif2ak2*. In the second stage, we co-transfected cells with siRNA mixtures, all including siRNA targeting both *Ifnb1* and *Eif2ak2*. Combined knockdown of *Ifnb1* and *Eif2ak2* resulted in a significant increase in cell survival compared to cells transfected with protein-encoding RNA only (p = 0.03), while adding siRNA targeting *Stat2* resulted in complete rescue of the cells (p<0.005), which continued to proliferate at a rate comparable to the mock-transfected control. Using this technique, we were able to transfect fibroblasts every 24 hours with RNA encoding Oct4, Sox2, Klf4, and Utf1, a combination of factors capable of reprogramming human fibroblasts to a pluripotent stem-cell state [31] (Fig. 2D). Many transfected cells expressed high levels of all four factors, and many mitotic cells were observed (Fig. 2E).

**Figure 3:**
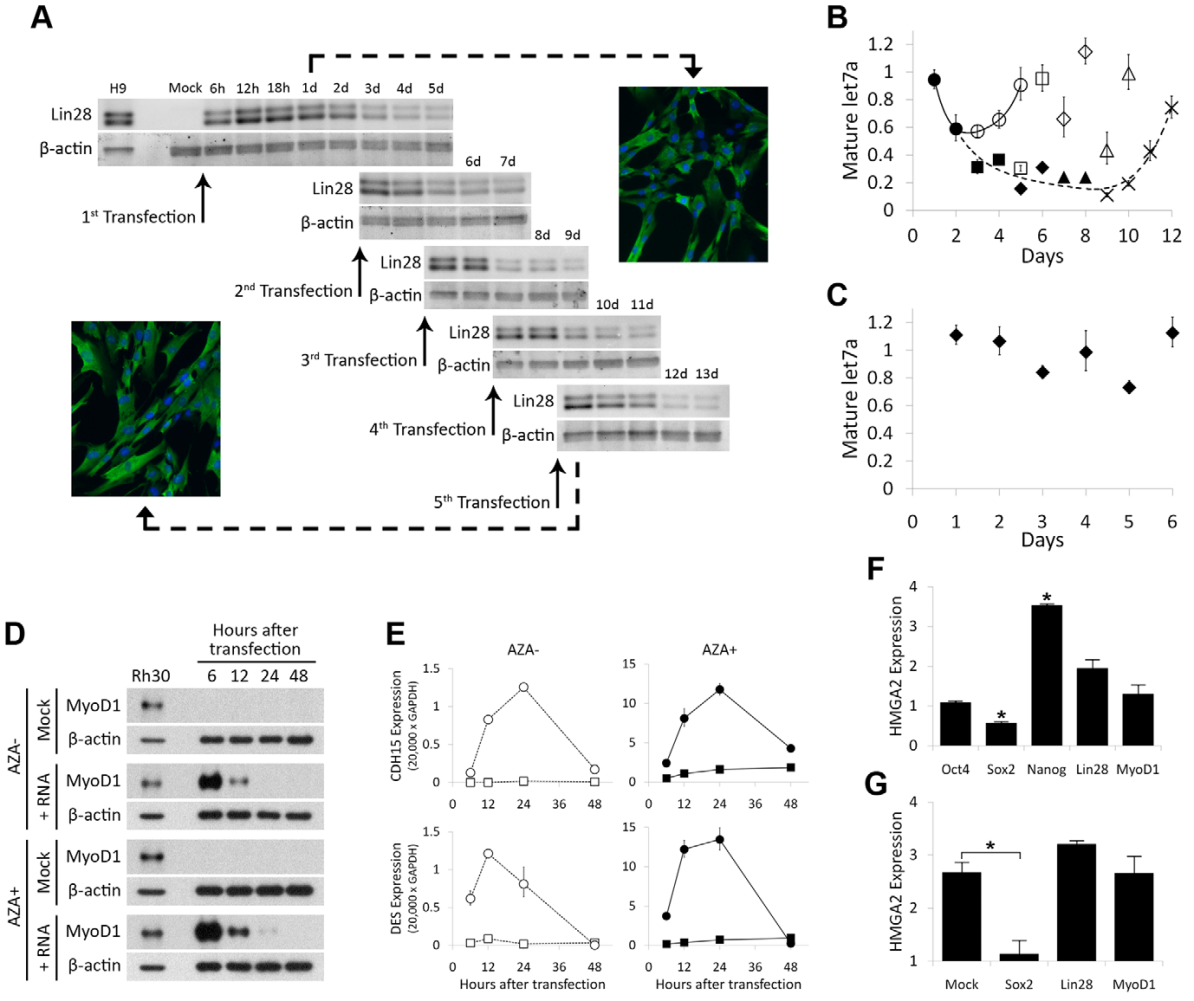
Repeated long-RNA transfection yields sustained, high-level expression of active proteins that modulate downstream targets. A. Sustaining high levels of Lin28 protein expression by frequent transfection with Lin28-encoding RNA. MRC-5 fibroblasts pre-transfected with a cocktail of siRNAs targeting *Ifnb1*, *Eif2ak2*, *Stat2*, and *Tlr3* were transfected five times with 0.5 µg of Lin28-encoding RNA and additional siRNA at 48-hour intervals. Cells were lysed at the indicated times, and the amount of Lin28 protein was analyzed by western blot. β-actin was used as a loading control. B. Sustained expression of Lin28 downregulates its target, mature let7 miRNA. Transfections were conducted as in (A). Data points indicate mature let7a levels in cells transfected once (circles), twice (squares), three times (diamonds), four times (triangles), or five times (crosses), relative to the level in mock-transfected cells. A solid smoothed line connects data points corresponding to cells transfected once, and a dashed smoothed line connects data points corresponding to cells transfected five times (dark symbols). U47 RNA was used as a loading control. Error bars indicate the standard error of replicate samples. C. let7a downregulation is Lin28-specific. Cells were transfected as in (A), but with MyoD1-encoding RNA. Error bars indicate the standard error of replicate samples. D. Expression of MyoD1 protein in fibroblasts. Fibroblasts cultured for three days with or without 2.5 µM 5-aza-dC (AZA) were electroporated with 1 µg/50 µL of MyoD1-encoding RNA. Cells were lysed at the indicated times, and the amount of MyoD1 protein in each sample was analyzed by western blot. E. Expression of MyoD1 in fibroblasts activates its normally silent targets, *Cdh15* and *Des* in a methylation-dependent manner. Cells were transfected as in (D), and expression of *Cdh15* and *Des* was measured by RT-PCR at the indicated times (squares, mock-transfected cells; circles, RNA-transfected cells). F. Regulation of *Hmga2* expression by reprogramming proteins. *Hmga2* expression in fibroblasts transfected with RNA encoding the indicated protein was measured by RT-PCR 24 hours after transfection. Values are given relative to mock-transfected cells. *Gapdh* was used as a loading control. *p<0.005. G. *Hmga2* expression in fibroblasts co-transfected with RNA encoding reprogramming proteins and a let7a inhibitor was measured by RT-PCR, 24 hours after transfection. Values are given relative to mock-transfected cells that received neither long RNA nor the let7a inhibitor. *Gapdh* was used as a loading control. *p = 0.004.

To determine whether long-RNA transfection could sustain high-level expression of biologically active protein through multiple rounds of cell division, we repeatedly transfected fibroblasts with siRNA targeting *Ifnb1*, *Eif2ak2*, *Stat2*, and *Tlr3*, and RNA encoding Lin28. Lin28 is a cytoplasmic, RNA-binding protein that is highly expressed both in embryonic stem cells, where it regulates cell growth [32], and in several cancers where it interferes with the maturation of members of the let7 family of miRNAs [33]–[35], which regulate *Hmga2* and downstream targets such as *Snai1* that promote metastasis and invasion [36]–[39]. We transfected fibroblasts five times at 48-hour intervals with Lin28-encoding RNA, and measured the levels of Lin28 protein and let7a miRNA (Fig. 3A). Lin28 protein was detected as early as six hours after the first transfection, remained highly expressed for two to three days, and was detected up to five days after each transfection. The level of let7a miRNA began to decrease two days after the first transfection, and in cells transfected only once, reached approximately 50% of the level in mock-transfected cells (p = 0.02), while in repeatedly transfected cells the level of let7a continued to decrease, reaching approximately 10% of the level in mock-transfected cells one day after the fifth transfection (p = 0.004) (Fig. 3B). Regardless of the number of transfections, let7a expression returned to normal levels approximately four days after the final transfection. The level of let7a miRNA in cells repeatedly transfected with MyoD1-encoding RNA remained within 70% of the level in mock-transfected cells, suggesting that the decrease in let7a expression following transfection with Lin28-encoding RNA was not a non-specific effect of long-RNA transfection (Fig. 3C). The observed decrease in the level of mature let7a miRNA in cells transfected five times over the course of ten days with Lin28-encoding RNA indicates both that each transfection was efficient, and that the translated protein was active.

We next used long-RNA transfection to express other known reprogramming proteins. Many of these proteins are transcription factors, which, unlike Lin28, must translocate to the nucleus and interact with genomic DNA to exert their function. We electroporated fibroblasts with RNA encoding the skeletal-muscle master gene MyoD1, and detected a high level of MyoD1 protein six hours after transfection (Fig. 3D). Two targets of MyoD1 that are normally silenced in fibroblasts, M-cadherin (*Cdh15*) and desmin (*Des*), were detected as early as six hours after transfection, and their expression peaked after 12–24 hours (Fig. 3E). Interestingly, expression of *Cdh15* and *Des* in cells treated with the demethylating agent 5-aza-dC showed similar dynamics, but reached a peak level ten times higher than in the untreated cells, suggesting that MyoD1-induced activation of *Cdh15* and *Des* in fibroblasts is inhibited by genomic methylation.

Because of the high transfection efficiency and activity of proteins expressed by long-RNA transfection, we hypothesized that this technique could be used to investigate early targets of reprogramming factors in somatic cells. Genes encoding pluripotent-stem-cell master regulators such as *Oct4* and *Nanog* are highly methylated in somatic cells, and as a result transient expression of proteins that promote transcription of these genes (Oct4, Sox2, and Nanog, for example) does not immediately cause their expression. Instead, somatic cell reprogramming may first require downregulation of somatic-cell genes, together with upregulation of ES-cell genes that are not completely silenced in somatic cells. One such gene, *Hmga2*, encodes a small chromatin-associated protein that cooperates with other factors to regulate gene expression. *Hmga2* is highly expressed in embryonic stem cells [40], young neural stem cells [41], and many human cancers, and is moderately expressed in various adult tissues including fibroblasts. Overexpressing *Hmga2* induces pituitary tumours in mice by binding to and inhibiting retinoblastoma protein [42], a tumour suppressor. *Hmga2*-induced pituitary adenomas exhibit >5-fold downregulation of *Sox2* compared with normal pituitary tissue [43]. We hypothesized that a reciprocal relationship might exist as a mechanism by which *Sox2*-expressing stem cells regulate *Hmga2* expression. We transfected fibroblasts with RNA encoding Oct4, Sox2, Nanog, Lin28 or MyoD1, and measured expression of *Hmga2* after 24 hours (Fig. 3F). As expected, cells transfected with RNA encoding Lin28 (which downregulates let7 miRNA, which itself downregulates *Hmga2*) showed slight overexpression of *Hmga2* (p = 0.03), while the level of *Hmga2* mRNA in cells transfected with RNA encoding Nanog was approximately 3.5 times that in mock-transfected cells (p = 0.002), and expression in cells transfected with RNA encoding Sox2 was approximately 0.5 times that in mock-transfected cells (p = 0.002). The high level of *Hmga2* expression in ES cells, combined with the upregulation of *Hmga2* observed in fibroblasts transfected with RNA encoding Nanog suggests that *Hmga2* may be an early downstream target of Nanog in fibroblasts during reprogramming.

To determine whether downregulation of *Hmga2* by Sox2 is sufficient to counteract *Hmga2* upregulation caused by inhibition of let7, we co-transfected cells with a let7-miRNA inhibitor and RNA encoding Sox2, and measured *Hmga2* expression after 24 hours (Fig. 3G). While cells transfected with only the let7 inhibitor showed approximately 3-fold upregulation of *Hmga2*, those transfected with both the inhibitor and Sox2-encoding RNA expressed *Hmga2* at the same level as mock-transfected cells, suggesting that an ES-cell level of Sox2 is sufficient to replace let7-mediated downregulation of *Hmga2*. The competing roles of Sox2, Nanog, and Lin28 in the regulation of *Hmga2* highlight the complex interactions between these factors that likely take place during reprogramming.

## Discussion

Long-RNA transfection is a versatile tool for investigating expression dynamics and protein interactions. In addition, the ability to maintain high-level expression of defined proteins in human cells for many days without genetic manipulation highlights the potential importance of long-RNA transfection in the development of reprogramming methods for therapeutic applications. Although techniques for in vitro synthesis of large quantities of capped, polyadenylated RNA have been available for some time [44], [45], as have a variety of delivery techniques including electroporation and lipid-mediated transfection [26], [46], the potent immune response triggered by long-RNA transfection has largely limited its use to studies of immunity, and has prevented the development of RNA-based reprogramming methods.

Here we have shown that combined knockdown of *Ifnb1*, *Eif2ak2*, and *Stat2* rescues human fibroblasts from the innate immune response triggered by frequent transfection with protein-encoding RNA, and enables sustained, high-level expression of active proteins. Interestingly, while we previously found that p53 knockdown alone increased the rate of recovery of cells transfected with long RNA [30], we now demonstrate that combined knockdown of *Ifnb1*, *Eif2ak2*, and *Stat2* is sufficient to allow frequent transfection with protein-encoding RNA, eliminating the need for p53 knockdown, which may facilitate the use of long-RNA transfection in therapeutic applications as p53 is crucial for the maintenance of genomic integrity.

Long-RNA transfection enables precise control over the timing and level of expression of encoded proteins. We used this characteristic to investigate the regulation of downstream targets of the cytoplasmic RNA-binding protein Lin28, and the transcription factor and skeletal-muscle master regulator MyoD1. In addition, we used long-RNA transfection to search for early targets of reprogramming factors in fibroblasts, and we provide evidence that the pluripotent-stem-cell master genes and reprogramming factors Sox2 and Nanog are novel upstream effectors of the proto-oncogene *Hmga2*.

Finally, while we have shown that siRNA-mediated immunosuppression alone is sufficient to enable frequent long-RNA transfection, the use of small-molecule immunosuppressants (for example, glucocorticoids such as cortisone [47] or dexamethasone), and/or protein immunosuppressants such as B18R, a vaccinia-virus-encoded soluble type I interferon receptor [48], [49], either alone or in combination with siRNA may increase the quantity of RNA that can be delivered to cells and the frequency of transfection, two parameters that will likely be critical in the design of efficient RNA-based reprogramming methods. The discovery that innate immune suppression enables frequent long-RNA transfection thus provides a clear path toward the development of culture and transfection protocols for RNA-based reprogramming.

## Materials and Methods

### Cell Culture

MEF cultures were prepared from E13 CF-1 mice (Charles River Laboratories) according to an approved protocol (MIT Committee on Animal Care #0307-023-10). Samples tested negative for mycoplasmal contamination by both DNA fluorochrome staining and live-culture methods. H9 human embryonic stem cells were obtained from the National Stem Cell Bank at passage 24, and were cultured on irradiated MEFs as described [50], [51]. Cells from frozen stocks (in hES-Cell Media +10% DMSO +30% Defined FBS) were seeded on plates coated with basement-membrane extract (Pathclear, Trevigen), and cultured in media conditioned for 24 hours on irradiated MEFs. Primary human fibroblasts from normal fetal lung tissue (MRC-5) or from normal adult skin (CCD-1109Sk) were obtained from the ATCC and were cultured according to their recommendations.

### In Vitro-Transcription

dsDNA templates were prepared as described [30]. Briefly, total RNA was extracted from H9 hES cells and enriched for poly(A)+ mRNA (Oligotex, Qiagen). Oct4, Sox2, Klf4, c-Myc, Utf1, Nanog, Lin28, MyoD1, and Aicda coding sequences, and β-globin UTRs were reverse transcribed using an RNase H- reverse transcriptase (MonsterScript, Epicentre). Template components were amplified with a high-fidelity polymerase (Phusion Hot Start, NEB or KAPA HiFi, Kapa Biosystems) and ligated with *E.coli* DNA ligase (NEB). Capped, poly(A)+ RNA was synthesized using the mScript mRNA Production System (Epicentre). The temperature and duration of the in vitro-transcription reaction were optimized for specificity and yield as described [30]. Transcripts were analyzed both before and after poly(A) tailing by denaturing formaldehyde-agarose gel electrophoresis. Primers used for assembly of in vitro-transcription templates are given in Table S2.

### Long-RNA Transfection

Lipid-mediated transfections (TransIT-mRNA, Mirus) were performed according to the manufacturer's instructions. Electroporation was performed as described [30]. Briefly, cells were trypsinized, washed once in Opti-MEM (Invitrogen), and resuspended in a total volume of 50 µL of Opti-MEM in a standard electroporation cuvette with a 2 mm gap. A 150uF capacitor charged to 150V was discharged into the cuvette to electroporate the cells. Warm media was added, and the cells were plated and cultured using standard protocols.

### Quantitative RT-PCR

TaqMan Gene Expression Assays (Applied Biosystems) were used in one-step RT-PCR reactions (iScript One-Step RT-PCR Kit, Bio-Rad) consisting of a 50C, 10 min reverse transcription step, followed by an initial denaturation step of 95C for 5 min, and 45 cycles of 95C for 15 sec and 55C for 30 sec.

### siRNA-Mediated Knockdown

Cells were electroporated in Opti-MEM containing the indicated siRNAs (Silencer Select or Anti-miR, Applied Biosystems), each at a final concentration of 100–800 nM (Table S1).

### Immunocytochemistry

Cells were rinsed in TBST and fixed for 10 minutes in 4% paraformaldehyde. Cells were then permeabilized for 10 minutes in 0.1% Triton X-100, blocked for 30 minutes in 1% casein, and incubated with appropriate antibodies (Table S3).

### Western Blot

Whole-cell lysates (Qproteome Mammalian Protein Prep Kit, Qiagen) were separated on a 12% polyacrylamide gel (ProSieve 50, Lonza) under reducing, denaturing conditions. Proteins were transferred onto a PVDF membrane (Immobilon-P, Millipore) in CAPS buffer, pH 11. Membranes were blocked in 5% skim milk, and probed with appropriate antibodies (Table S3). β-actin was used as a loading control.

## Supporting Information

Table S1 Concentrations of siRNA used in the combinatorial screen.
(0.03 MB DOC)

Table S2 Primers for in vitro-transcription template assembly.
(0.03 MB DOC)

Table S3 Antibodies.
(0.03 MB DOC)

## References

[1] Takahashi K, Yamanaka S (2006). Induction of pluripotent stem cells from mouse embryonic and adult fibroblast cultures by defined factors.. Cell.

[2] Okita K, Ichisaka T, Yamanaka S (2007). Generation of germline-competent induced pluripotent stem cells.. Nature.

[3] Wernig M, Meissner A, Foreman R, Brambrink T, Ku M (2007). In vitro reprogramming of fibroblasts into a pluripotent ES-cell-like state.. Nature.

[4] Takahashi K, Tanabe K, Ohnuki M, Narita M, Ichisaka T (2007). Induction of pluripotent stem cells from adult human fibroblasts by defined factors.. Cell.

[5] Yu J, Vodyanik MA, Smuga-Otto K, Antosiewicz-Bourget J, Frane JL (2007). Induced pluripotent stem cell lines derived from human somatic cells.. Science.

[6] Park IH, Zhao R, West JA, Yabuuchi A, Huo H (2008). Reprogramming of human somatic cells to pluripotency with defined factors.. Nature.

[7] Lowry WE, Richter L, Yachechko R, Pyle AD, Tchieu J (2008). Generation of human induced pluripotent stem cells from dermal fibroblasts.. Proc Natl Acad Sci U S A.

[8] Yu J, Hu K, Smuga-Otto K, Tian S, Stewart R (2009). Human induced pluripotent stem cells free of vector and transgene sequences.. Science.

[9] Jia F, Wilson KD, Sun N, Gupta DM, Huang M A nonviral minicircle vector for deriving human iPS cells.. Nat Methods.

[10] Zhou H, Wu S, Joo JY, Zhu S, Han DW (2009). Generation of induced pluripotent stem cells using recombinant proteins.. Cell Stem Cell.

[11] Kim D, Kim CH, Moon JI, Chung YG, Chang MY (2009). Generation of human induced pluripotent stem cells by direct delivery of reprogramming proteins.. Cell Stem Cell.

[12] Yoneyama M, Kikuchi M, Natsukawa T, Shinobu N, Imaizumi T (2004). The RNA helicase RIG-I has an essential function in double-stranded RNA-induced innate antiviral responses.. Nat Immunol.

[13] Hornung V, Ellegast J, Kim S, Brzozka K, Jung A (2006). 5′-Triphosphate RNA is the ligand for RIG-I.. Science.

[14] Saito T, Owen DM, Jiang F, Marcotrigiano J, Gale M (2008). Innate immunity induced by composition-dependent RIG-I recognition of hepatitis C virus RNA.. Nature.

[15] Takahasi K, Yoneyama M, Nishihori T, Hirai R, Kumeta H (2008). Nonself RNA-sensing mechanism of RIG-I helicase and activation of antiviral immune responses.. Mol Cell.

[16] Yoneyama M, Fujita T (2008). Structural mechanism of RNA recognition by the RIG-I-like receptors.. Immunity.

[17] Schmidt A, Schwerd T, Hamm W, Hellmuth JC, Cui S (2009). 5′-triphosphate RNA requires base-paired structures to activate antiviral signaling via RIG-I.. Proc Natl Acad Sci U S A.

[18] Schlee M, Roth A, Hornung V, Hagmann CA, Wimmenauer V (2009). Recognition of 5′ triphosphate by RIG-I helicase requires short blunt double-stranded RNA as contained in panhandle of negative-strand virus.. Immunity.

[19] Alexopoulou L, Holt AC, Medzhitov R, Flavell RA (2001). Recognition of double-stranded RNA and activation of NF-kappaB by Toll-like receptor 3.. Nature.

[20] Kariko K, Ni H, Capodici J, Lamphier M, Weissman D (2004). mRNA is an endogenous ligand for Toll-like receptor 3.. J Biol Chem.

[21] Kariko K, Buckstein M, Ni H, Weissman D (2005). Suppression of RNA recognition by Toll-like receptors: the impact of nucleoside modification and the evolutionary origin of RNA.. Immunity.

[22] Diebold SS, Kaisho T, Hemmi H, Akira S, Reis e Sousa C (2004). Innate antiviral responses by means of TLR7-mediated recognition of single-stranded RNA.. Science.

[23] Das HK, Das A, Ghosh-Dastidar P, Ralston RO, Yaghmai B (1981). Protein synthesis in rabbit reticulocytes. Purification and characterization of a double-stranded RNA-dependent protein synthesis inhibitor from reticulocyte lysates.. J Biol Chem.

[24] Levin DH, Petryshyn R, London IM (1981). Characterization of purified double-stranded RNA-activated eIF-2 alpha kinase from rabbit reticulocytes.. J Biol Chem.

[25] Bode JG, Brenndorfer ED, Haussinger D (2007). Subversion of innate host antiviral strategies by the hepatitis C virus.. Arch Biochem Biophys.

[26] Malone RW, Felgner PL, Verma IM (1989). Cationic liposome-mediated RNA transfection.. Proc Natl Acad Sci U S A.

[27] Kozak M (1986). Point mutations define a sequence flanking the AUG initiator codon that modulates translation by eukaryotic ribosomes.. Cell.

[28] Russell JE, Liebhaber SA (1996). The stability of human beta-globin mRNA is dependent on structural determinants positioned within its 3′ untranslated region.. Blood.

[29] Yu J, Russell JE (2001). Structural and functional analysis of an mRNP complex that mediates the high stability of human beta-globin mRNA.. Mol Cell Biol.

[30] Angel M (2008). Extended Transient Transfection by Repeated Delivery of In Vitro-Transcribed RNA [Master's Thesis]..

[31] Zhao Y, Yin X, Qin H, Zhu F, Liu H (2008). Two supporting factors greatly improve the efficiency of human iPSC generation.. Cell Stem Cell.

[32] Xu B, Zhang K, Huang Y (2009). Lin28 modulates cell growth and associates with a subset of cell cycle regulator mRNAs in mouse embryonic stem cells.. RNA.

[33] Viswanathan SR, Daley GQ, Gregory RI (2008). Selective blockade of microRNA processing by Lin28.. Science.

[34] Rybak A, Fuchs H, Smirnova L, Brandt C, Pohl EE (2008). A feedback loop comprising lin-28 and let-7 controls pre-let-7 maturation during neural stem-cell commitment.. Nat Cell Biol.

[35] Heo I, Joo C, Cho J, Ha M, Han J (2008). Lin28 mediates the terminal uridylation of let-7 precursor MicroRNA.. Mol Cell.

[36] Mayr C, Hemann MT, Bartel DP (2007). Disrupting the pairing between let-7 and Hmga2 enhances oncogenic transformation.. Science.

[37] Lee YS, Dutta A (2007). The tumor suppressor microRNA let-7 represses the HMGA2 oncogene.. Genes Dev.

[38] Dangi-Garimella S, Yun J, Eves EM, Newman M, Erkeland SJ (2009). Raf kinase inhibitory protein suppresses a metastasis signalling cascade involving LIN28 and let-7.. EMBO J.

[39] Shell S, Park SM, Radjabi AR, Schickel R, Kistner EO (2007). Let-7 expression defines two differentiation stages of cancer.. Proc Natl Acad Sci U S A.

[40] Li O, Vasudevan D, Davey CA, Droge P (2006). High-level expression of DNA architectural factor HMGA2 and its association with nucleosomes in human embryonic stem cells.. Genesis.

[41] Nishino J, Kim I, Chada K, Morrison SJ (2008). Hmga2 promotes neural stem cell self-renewal in young but not old mice by reducing p16Ink4a and p19Arf Expression.. Cell.

[42] Fedele M, Visone R, De Martino I, Troncone G, Palmieri D (2006). HMGA2 induces pituitary tumorigenesis by enhancing E2F1 activity.. Cancer Cell.

[43] De Martino I, Visone R, Palmieri D, Cappabianca P, Chieffi P (2007). The Mia/Cd-rap gene expression is downregulated by the high-mobility group A proteins in mouse pituitary adenomas.. Endocr Relat Cancer.

[44] Paterson BM, Rosenberg M (1979). Efficient translation of prokaryotic mRNAs in a eukaryotic cell-free system requires addition of a cap structure.. Nature.

[45] Melton DA, Krieg PA, Rebagliati MR, Maniatis T, Zinn K (1984). Efficient in vitro synthesis of biologically active RNA and RNA hybridization probes from plasmids containing a bacteriophage SP6 promoter.. Nucleic Acids Res.

[46] Zabner J, Fasbender AJ, Moninger T, Poellinger KA, Welsh MJ (1995). Cellular and molecular barriers to gene transfer by a cationic lipid.. J Biol Chem.

[47] Kilbourne ED, Smart KM, Pokorny BA (1961). Inhibition by cortisone of the synthesis and action of interferon.. Nature.

[48] Symons JA, Alcami A, Smith GL (1995). Vaccinia virus encodes a soluble type I interferon receptor of novel structure and broad species specificity.. Cell.

[49] Colamonici OR, Domanski P, Sweitzer SM, Larner A, Buller RM (1995). Vaccinia virus B18R gene encodes a type I interferon-binding protein that blocks interferon alpha transmembrane signaling.. J Biol Chem.

[50] Thomson JA, Itskovitz-Eldor J, Shapiro SS, Waknitz MA, Swiergiel JJ (1998). Embryonic stem cell lines derived from human blastocysts.. Science.

[51] Watanabe K, Ueno M, Kamiya D, Nishiyama A, Matsumura M (2007). A ROCK inhibitor permits survival of dissociated human embryonic stem cells.. Nat Biotechnol.

