# Supplementary material for: Innate Immune Suppression Enables Frequent Transfection with RNA Encoding Reprogramming Proteins
Source: PLoS One. 2010 Jul 23;5(7):e11756. doi: 10.1371/journal.pone.0011756 (PMC2909252; doi:10.1371/journal.pone.0011756)
Supplement: Table S1 — Concentrations of siRNA used in the combinatorial screen. (0.03 MB DOC) [file pone.0011756.s001.doc]

Stage 1:

| Target | Final Concentration/nM |
| --- | --- |
| Ifnb1 | 200 |
| Tlr3 | 200 |
| Rarres3 | 200 |
| Eif2ak2 | 200 |
| Stat1 | 200 |
| Stat2 | 200 |
| Tp53 | 800 |
| Cdkn1a | 200 |

Stage 2:

| Target | Final Concentration/nM | Total Concentration/nM |
| --- | --- | --- |
| I+E | 200+200 | 400 |
| I+E+T3 | 200+200+200 | 600 |
| I+E+S2 | 200+200+200 | 600 |
| I+E+T3+S2 | 200+200+200+200 | 800 |
| I+E+T3+S1+S2 | 200+200+200+100+200 | 900 |
| I+E+T3+S2+TP+C | 200+200+200+200+800+200 | 1800 |
| I+E+T3+S1+S2+TP+C+R | 200+200+200+100+200+800+200+100 | 2000 |
