# Supplementary material for: Innate Immune Suppression Enables Frequent Transfection with RNA Encoding Reprogramming Proteins
Source: PLoS One. 2010 Jul 23;5(7):e11756. doi: 10.1371/journal.pone.0011756 (PMC2909252; doi:10.1371/journal.pone.0011756)
Supplement: Table S2 — Primers for in vitro-transcription template assembly. (0.03 MB DOC) [file pone.0011756.s002.doc]

HBB 5-UTR Forward Primer: taatacgactcactatagggacatttgcttctgacacaactgtg

HBB 5-UTR Kz Reverse Primer: aaaaagctagctgtttgaggttgctagtgaacacagttgtg

HBB 3-UTR Forward Primer: aaaaaaccggtgctcgctttcttgctgtc

HBB 3-UTR Reverse Primer: gcaatgaaaataaatgttttttattaggcagaatccagatg

Oct4 CDS Kz Forward Primer: aaaaagctagccaccatggcgggacacctg

Oct4 CDS Reverse Primer: aaaaaaccggttcagtttgaatgcatgggagag

Sox2 CDS Kz Forward Primer: aaaaagctagccgccaccatgtacaacatgatg

Sox2 CDS Reverse Primer: aaaaaaccggttcacatgtgtgagaggggcag

Klf4 CDS Kz 2 Forward Primer: aaaaagctagccaccatgaggcagccacctg

Klf4 CDS Reverse Primer: aaaaaaccggtttaaaaatgcctcttcatgtgtaaggcgag

UTF1 CDS Kz 3 Forward Primer: aaaaagctagccaccatgctgctccggccccgcag

UTF1 CDS 2 Reverse Primer: aaaaaaccggttcactggcacgggtccctg

Nanog CDS Kz Forward Primer: aaaaagctagccaccatgagtgtggatccag

Nanog CDS Reverse Primer: aaaaaaccggttcacacgtcttcaggttgcatg

Lin28 CDS Kz Forward Primer: aaaaagctagccaccatgggctccgtgtccaac

Lin28 CDS Reverse Primer: aaaaaaccggttcaattctgtgcctccgggag

AICDA CDS Kz Forward Primer: aaaaagctagccaccatggacagcctcttgatg

AICDA CDS Reverse Primer: aaaaaaccggttcaaagtcccaaagtacgaaatgc

MyoD1 CDS Kz Forward Primer: aaaaagctagccaccatggagctactgtcgccac

MyoD1 CDS Reverse Primer: aaaaaaccggttcagagcacctggtatatcgggttg

Yellow: T7 promoter

Red: Restriction site

Green: Kozak sequence
