# Supplementary material for: Innate Immune Suppression Enables Frequent Transfection with RNA Encoding Reprogramming Proteins
Source: PLoS One. 2010 Jul 23;5(7):e11756. doi: 10.1371/journal.pone.0011756 (PMC2909252; doi:10.1371/journal.pone.0011756)
Supplement: Table S3 — Antibodies. (0.03 MB DOC) [file pone.0011756.s003.doc]

| Antigen | Application | Antibody | Vendor |
| --- | --- | --- | --- |
| Oct4 | Immunocytochemistry, Western Blot | ab27985 | Abcam |
|  | Immunocytochemistry | MAB1759 | R&D |
| Sox2 | Immunocytochemistry, Western Blot | AB5603 | Millipore |
| Klf4 | Immunocytochemistry | AF3640 | R&D |
| Utf1 | Immunocytochemistry | MAB4337 | Millipore |
| Nanog | Immunocytochemistry, Western Blot | N3038 | Sigma |
| Lin28 | Immunocytochemistry, Western Blot | ab46020 | Abcam |
| MyoD1 | Immunocytochemistry | IMG-132 | Imgenex |
|  | Western Blot | ab16148 | Abcam |
| Actb | Western Blot | ab8226 | Abcam |
